# Revisiting infrageneric concepts in *Geastrum* (Geastraceae, Geastrales) with three new species

**DOI:** 10.3897/mycokeys.131.181414

**Published:** 2026-05-04

**Authors:** Tian Wang, Qin Na, Li Zou, Hui Zeng, Yupeng Ge

**Affiliations:** 1 School of Horticulture, Ludong University, Yantai 264025, China Ludong University Yantai China; 2 College of Forestry, Northeast Forestry University, Harbin 150040, China Fujian Academy of Agricultural Sciences Fuzhou China; 3 Institute of Edible Fungi, Fujian Academy of Agricultural Sciences, Fuzhou 350014, China Northeast Forestry University Harbin China; 4 National and Local Joint Engineering Research Center for Breeding & Cultivation of Features Edible Fungi, Fuzhou 350014, China National and Local Joint Engineering Research Center for Breeding & Cultivation of Features Edible Fungi Fuzhou China

**Keywords:** Gasteroid fungi, new taxon, systematics, taxonomy

## Abstract

*Geastrum* is a typical saprotrophic genus with nearly 130 species distributed worldwide, yet its infrageneric classification has traditionally relied on morphological characteristics whose phylogenetic significance remains uncertain. Here, we revisit infrageneric concepts in *Geastrum* using Chinese material, integrating detailed morphological observations with multi-locus phylogenetic analyses. Eight specimens collected from temperate forests in Northeast and East China yielded three new species: *G. capillitiononramosum***sp. nov**., *G. villopannosum***sp. nov**., and *G. kunyuense***sp. nov**. These species share a sessile endoperidial body lacking a collar and apophysis, a distinctly delimited fibrillose peristome, and globose to subglobose basidiospores with columnar ornamentation but differ mainly in the structure of the mycelial layer and the branching and ornamentation of capillitial hyphae. Phylogenetic analyses based on a concatenated ITS–nrLSU–*rpb1* dataset recovered 12 well-supported clades and confirmed the distinct phylogenetic positions of the three new species. The resulting topology further demonstrates that several traditional infrageneric groupings defined by key morphological traits, such as the hygroscopic behavior of the exoperidium and debris-encrusted mycelial layers, are only partially congruent with molecular relationships, reflecting repeated character evolution across distant lineages. Nevertheless, specific combinations of peristome structure, mycelial layer features, and capillitial morphology remain informative at the species level. With the descriptions of three new species and an updated key to 43 Chinese taxa, this study refines current knowledge of *Geastrum* diversity in China and provides new evidence for reassessing infrageneric classification within the genus.

## Introduction

*Geastrum* Pers. is readily recognized by its exoperidium, normally splitting into a stellate pattern at maturity (Persoon 1794; Staněk 1958; Sunhede 1989; Zamora et al. 2014a). It is the type and largest genus of Geastraceae Corda and has long been accepted at the generic rank (Fries 1829; De Toni 1887; Sunhede 1989; Lloyd 1902; Zhou et al. 2007; Kirk et al. 2008). The genus was erected by Persoon (1794), with only three species originally included: *G. rufescens* Pers., *G. quadrifidum* Pers., and *G. multifidum* Pers. Although over 388 names representing more than 200 taxa are currently deposited in Index Fungorum (accessed on 17 February 2026), only 128 species have been formally described morphologically (Zhou et al. 2007; Gube et al. 2009; Hemmes et al. 2011; Jeppson et al. 2013; Zamora et al. 2014a; Da Silva Alfredo et al. 2016; He et al. 2019, 2024). A fibrillose or fimbriate peristome, the presence or absence of a stalk, the shapes and hygroscopic behavior of the exoperidium, the ability of the mycelial layer to encrust debris, the occurrence of rhizomorphs, the growth habit, the microstructure of the mycelial layer, and specific chemical reactions were the key characters of *Geastrum* (Fries 1829; De Toni 1887; Lloyd 1902; Dissing and Lange 1962; Ponce de León 1968; Sunhede 1989; Zhou et al. 2007; Zamora et al. 2014a). During the past 200 years, species of the genus have been found in the Southern and Northern Hemispheres, mainly in South America, Europe, and North America, with fewer findings in Asia, Africa, and Oceania (Cunningham 1944; Staněk 1958; Dissing and Lange 1962; Ponce de León 1968; Sunhede 1989; Soto and Wright 2000; Zhou et al. 2007; Jeppson et al. 2013; Zamora et al. 2013, 2014a, 2014b, 2014c, 2015; Da Silva Alfredo et al. 2016; Crous et al. 2018, 2023).

The infrageneric classification of *Geastrum* has been repeatedly revised due to the increasing number of known species and the diversity of morphological characteristics (De Toni 1887; Lloyd 1902; Staněk 1958; Dissing and Lange 1962; Ponce de León 1968; Jeppson et al. 2013; Zamora et al. 2014a). Initially, *Geastrum* was divided into seven sections based on the characters of the exoperidium, the shapes of the peristome, and a stalked or sessile endoperidial body (De Toni 1887). Based on the hygroscopic behavior of the exoperidium, Lloyd (1902) proposed a revised infrageneric system and recognized two additional sections. Based on numerous specimens from Europe, Staněk (1958) emphasized the debris–encrusting mycelial layer as a key diagnostic character and erected two new sections: sect. *Perimyceliata* V. J. Staněk (two subsections and six stirps) and sect. *Basimyceliata* V. J. Staněk (two subsections and three stirps). Sect. *Basimyceliata* was revised into three subsections and eight stirps (including two new subsections and five new stirps) by Dissing and Lange (1962) based on Congolese specimens, according to the characters of growth habit. Subsequently, five new sections, two new subsections, and 15 new groups were proposed by Ponce de León (1968) through a combination of the results of Staněk (1958) and Dissing and Lange (1962). Until 2013, *Geastrum* was divided into 11 clades by Jeppson, and it was proposed that the hygroscopic behavior of the exoperidium should not be the key character for dividing the genus (De Toni 1887; Lloyd 1902; Staněk 1958; Dissing and Lange 1962; Ponce de León 1968; Jeppson et al. 2013). With the identification of 23 clades and the proposal of seven new sections and four new subsections, a new classification of *Geastrum* was established by Zamora et al. (2014a) based on a multi-locus (ITS, nrLSU, *rpb1*, and *atp6*) dataset. According to Zamora et al. (2014), phylogenetic analyses failed to support previous infrageneric classifications of *Geastrum* that were based on key morphological characters defining subgenera, sections, subsections, stirps, and groups (De Toni 1887; Lloyd 1902; Staněk 1958; Dissing and Lange 1962; Ponce de León 1968; Zamora et al. 2014a). These findings indicate that infrageneric concepts within *Geastrum* remain unresolved and warrant further evaluation using integrated morphological and molecular evidence from expanded geographic sampling.

In China, 43 species of *Geastrum*, comprising 27 known species and 16 new species, have been reported to date (Teng 1963; Tai 1979; Liu 1984; Zhou et al. 2007; Han et al. 2016; Zhou et al. 2022; Wang et al. 2023; Yang et al. 2025). Among these, 15 species have been reported in the Northwest and Southwest regions, 10 and six species in the North and Northeast areas, respectively, and fewer than five species in the East, South, and Central regions (Teng 1963; Tai 1979; Liu 1984; Zhou et al. 2007; Han et al. 2016; Zhou et al. 2022; Wang et al. 2023; Yang et al. 2025). Given the broad bioclimatic range of China and its representation of multiple infrageneric lineages, materials from this region are particularly valuable for evaluating infrageneric classification in *Geastrum*. During our ongoing research on gasteroid fungi in China, we describe three new species of *Geastrum* from typical temperate forests in Northeast and East China and integrate morphological and molecular evidence to reassess the distribution and taxonomic utility of key infrageneric characters.

## Materials and methods

### Specimen collection and morphological study

Specimens were collected from Heilongjiang, Jilin, and Shandong provinces in China. During fieldwork, high-definition habitat photographs of fresh basidiomata were taken using an Olympus E-M1 Mark III (Olympus, Tokyo, Japan), a Sony ILCE-A7M3 (Sony, Tokyo, Japan) camera, M. Zuiko Digital ED 12–40 mm, 60 mm lenses (Olympus, Tokyo, Japan), and a Sigma 70 mm f2.8 EX DG MACRO lens (Sigma, Kobe, Japan). Detailed information for each numbered specimen was recorded, including size, color, and shapes of the pseudoparenchymatous layer, fibrous layer, mycelial layer, endoperidium, and peristome. The nomenclature of color descriptions follows Ridgway (1912). Small tissue pieces were taken for molecular material. Following complete drying in a 40 °C electric oven (Stöckli, Netstal), the specimens were placed in Ziploc bags along with allochroic silica gel. The voucher specimens were preserved in the fungarium of the Fujian Academy of Agricultural Sciences (FFAAS), China.

Microscopic characteristics were observed using a Lab A1 microscope (Carl Zeiss AG, Jena, Germany). Dried specimens were rehydrated in a 5% KOH aqueous solution and stained with a 1% Congo Red solution when necessary. Microstructures were photographed and measured using ZEN 2.3 (Blue Edition) (Carl Zeiss Microscopy GmbH, Jena, Germany). For each specimen, 25 mature basidiospores were randomly selected and measured. The results were presented [a/b/c] (d)e–***f***–g(h) × (i)j–***k***–l(m) µm, [Q = (n)o–p(q), Qm = ***r*** ± s]. In this notation, a, b, and c were the numbers of measured basidiospores, basidiomata, and specimens, respectively; d and h were the minimum and maximum lengths (5% extremum); e–g represented the range of at least 90% of values; f was the average length; width (i–m) was expressed in the same manner (Na et al. 2022, 2024). Q represented the length-to-width ratio of the basidiospores, and Qm = the mean value of all basidiospore Q ratios ± the sample standard deviation (Bandala and Montoya 2000; Dramani et al. 2020; Na et al. 2021). Additionally, for each specimen, at least 20 measurements of the diameter were randomly conducted for each of the following: capillitial hyphae, pseudoparenchymatous layer cells, fibrous layer hyphae, and mycelial layer hyphae.

Based on habitat photographs, field records, and microscopic observations, illustrations of the basidiomata and microstructures were prepared. The line drawings were subsequently scanned as TIFF files using a Canon LiDE 120 scanner (Canon, Tokyo, Japan). Finally, the scanned images were imported into Photoshop CC 2018.

### DNA extraction and PCR amplification

Genomic DNA was extracted from each sample using a NuClean PlantGen DNA kit (Kangwei Century Biotechnology Co., Beijing, China). Three gene fragments, ITS (the internal transcribed spacer), nrLSU (the largest subunit of nuclear ribosomal RNA), and *rpb1* (the largest subunit of RNA polymerase II), were amplified. The primers were ITS1/ITS4, LR0R/LR7, and *RPB1*GEA-1F/*RPB1*GEA-2r (White et al. 1990; Hopple and Vilgalys 1999; Zamora et al. 2014a). Amplifications were performed in a final volume of 25 μL, containing 12.5 μL of 2 × Utaq PCR MasterMix (ZomanBio, Beijing, China), 8.5 μL of ddH_2_O, 1 μL of each primer, and 2 μL of DNA template. The PCR amplification for ITS was 94 °C for 4 min; then 34 cycles of 94 °C for 45 s, 52 °C for 45 s, and 72 °C for 1 min, with a final extension at 72 °C for 10 min (White et al. 1990). For nrLSU, PCR amplification was performed under the following conditions: 95 °C for 5 min, followed by 30 cycles of 95 °C for 30 s, 55 °C for 30 s, and 72 °C for 1 min, with a final extension at 72 °C for 10 min ( Hopple and Vilgalys 1999). The *rpb1* gene was amplified using the following PCR program: 94 °C for 5 min; then 12 cycles of 94 °C for 30 s, 57 °C for 30 s (decreasing by 0.5 °C per cycle), and 72 °C for 90 s; subsequently, 23 cycles of 94 °C for 30 s, 53 °C for 30 s, and 72 °C for 90 s; with a final extension at 72 °C for 10 min. The PCR products were sequenced using the dideoxy termination method by the Beijing Genomics Institute (Beijing, China). Sequence quality was checked, and alignments were edited using BioEdit 7.2.5.0 software. The pBLUE-T kit (Beijing Zoman Biotechnology Co., Beijing, China) was used to obtain high-quality sequences.

### Phylogenetic tree construction

Phylogenetic trees were reconstructed using both Bayesian inference (BI) and maximum likelihood (ML) methods. Following a BLAST search, homologous sequences (nucleotide identity above 90%) of self-sequenced data were downloaded from the NCBI database (https://www.ncbi.nlm.nih.gov/). Sequences were aligned using MAFFT v7.520 with the auto strategy (FFT-NS-1, FFT-NS-2, FFT-NS-i, or L-INS-i), and missing data were treated as gaps (Katoh et al. 2002; Tamura et al. 2011). Sequences were manually aligned and adjusted with BioEdit 7.2.5.0 (Hall 1999). For BI analysis, the best-fit nucleotide substitution model for each gene fragment was determined separately using MrModelTest 2.3 (Nylander 2004). Subsequently, MrBayes 3.2.6 was used to run the analysis with five chains for 5,000,000 MCMC generations, sampling every 1,000 generations (Ronquist et al. 2003). With the average deviation dropping below 0.01 at the end of the run, the first 25% of trees were discarded as burn-in, and the results were output with the “sump burnin” and “sumt burnin” commands (Ronquist et al. 2003). Tracer v1.7.2 was used to verify the effective sample size (ESS) in Bayesian inference (BI) analysis (Rambaut et al. 2018). The ML phylogenetic analysis was performed in raxmlGUI v2.0 (Edler et al. 2021). The phylogenetic tree was visualized with FigTree v1.4.3. The final editing of the trees was completed in Adobe Illustrator 2020.

## Results

### Phylogenetic analyses

**Figure 1.**
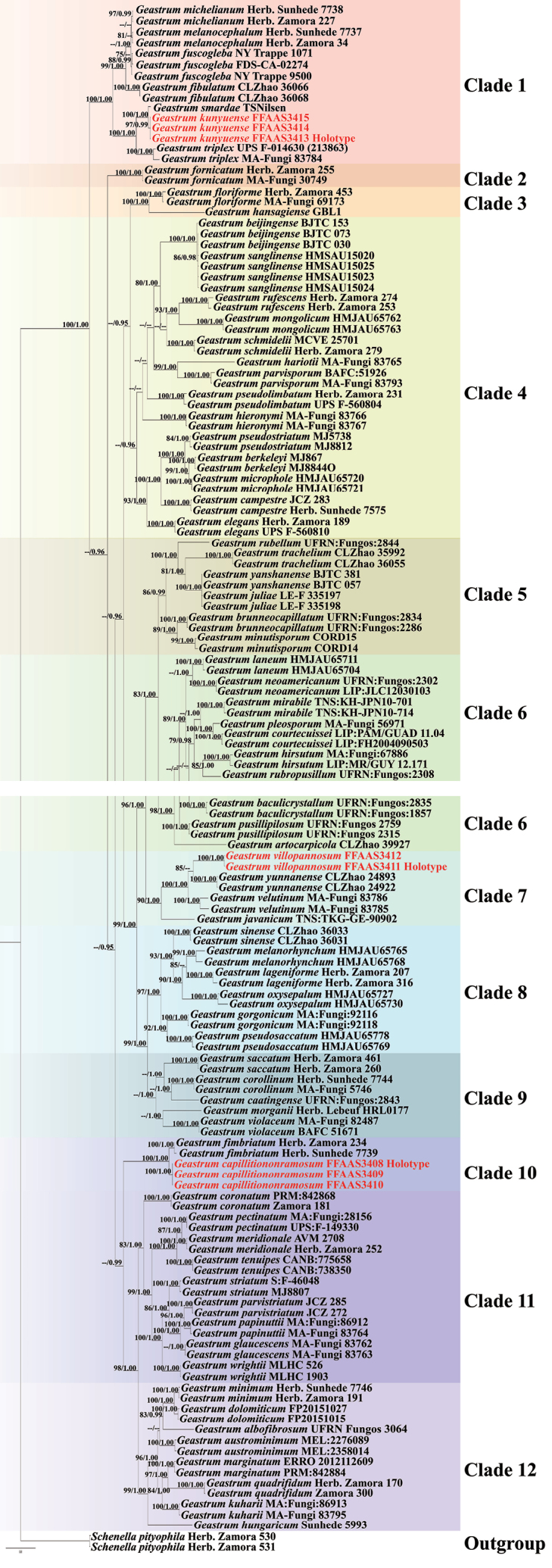
Multi-locus phylogenetic tree inferred from Bayesian inference (BI) analysis based on the ITS + nrLSU + *rpb1* dataset. Branches with maximum likelihood bootstrap values equal to or over 75% and Bayesian posterior probability values equal to or over 0.95 are indicated. Different clades are marked with different colors. The new species are indicated in red.

The multi-gene sequence dataset comprised 361 sequences (144 ITS, 130 nrLSU, and 87 *rpb1*), of which 340 were downloaded from GenBank and 21 were newly obtained in this study. Detailed information is provided in Table 1. The aligned dataset contained 2728 sites, including gaps. The lengths of the aligned regions were as follows: ITS1 for 295 bp, 5.8S for 158 bp, ITS2 for 329 bp, nrLSU for 909 bp, and *rpb1* for 1037 bp (*rpb1* exons for 385 bp and *rpb1* introns for 652 bp). Among the 21 newly generated sequences, one degenerate base was detected. For BI analysis, the best-fit nucleotide substitution models for ITS1 and ITS2 were GTR+G and HKY+I+G, respectively; 5.8S, nrLSU, *rpb1* exons, and *rpb1* introns were all GTR+I+G. For ML phylogenetic analysis, the optimal models for ITS1, 5.8S, ITS2, and nrLSU were JC, JC, K80+G, and TN93+I+G, respectively. For *rpb1* exons and introns, the optimal models were TIM1+G and TIM2+G, respectively. In the BI analysis, the average deviation of split frequencies was 0.002636, the ESS was 1339.3, and the average potential scale reduction factor (PSRF) was 1.000, after running 5,000,000 generations of MCMC. A nonparametric bootstrap analysis with 1,000 replicates was conducted using the maximum-likelihood phylogeny, which had an optimized log-likelihood of -28366.895371. The BI and ML trees exhibited similar topologies, and the BI tree was selected and displayed (Fig. 1). Bootstrap values (BS) greater than 75 and Bayesian posterior probabilities (BPP) greater than 0.95 were shown on each branch node.

**Table 1.** Names, vouchers, localities, references, and corresponding GenBank accession numbers of the taxa used in the phylogenetic analysis.

Species	Voucher/strain no.	Locality	GenBank no.			Reference
			ITS	nrLSU	*rpb1*	
*G. albofibrosum*	UFRN Fungos 3064	Brazil	NR_189922	NG_243171	—	Unpublished
*G. artocarpicola*	CLZhao 39927	China	PQ484150	—	—	Yang et al. 2025
*G. austrominimum*	MEL:2276089	Australia	KP687490	KP687451	KP687532	Zamora et al. 2015
*G. austrominimum*	MEL:2358014	Australia	KP687492	KP687453	KP687534	Zamora et al. 2015
*G. baculicrystallum*	UFRN:Fungos:2835	Brazil	MH634995	MH635028	—	Accioly et al. 2019
*G. baculicrystallum*	UFRN:Fungos:1857	Brazil	MH635018	MH635035	—	Accioly et al. 2019
*G. beijingense*	BJTC 153	China	MZ508875	MZ509379	MZ571170	Zhou et al. 2022
*G. beijingense*	BJTC 073	China	MZ508873	MZ509377	MZ571168	Zhou et al. 2022
*G. beijingense*	BJTC 030	China	MZ508874	MZ509378	MZ571169	Zhou et al. 2022
*G. berkeleyi*	MJ867	Slovakia	KC581985	KC581985	—	Jeppson et al. 2013
*G. berkeleyi*	MJ8844O	Sweden	KC581988	KC581988	—	Jeppson et al. 2013
*G. brunneocapillatum*	UFRN:Fungos:2834	Brazil	MH634997	MH635030	—	Accioly et al. 2019
*G. brunneocapillatum*	UFRN:Fungos:2286	Brazil	MH634996	MH635029	—	Accioly et al. 2019
*G. caatingense*	UFRN:Fungos:2843	Brazil	MH253884	MH253886	—	Unpublished
*G. campestre*	JCZ 283	Spain	JN943167	JN939575	JN991286	Unpublished
*G. campestre*	Herb. Sunhede 7575	Sweden	KF988357	KF988479	KF988614	Zamora et al. 2014a
***G. capillitiononramosum***	***FFAAS3408* Holotype**	**China**	**PX656672**	**PX656680**	**PX676540**	**This study**
***G. capillitiononramosum***	***FFAAS3409***	**China**	**PX656673**	**PX656681**	**PX676541**	**This study**
***G. capillitiononramosum***	***FFAAS3410***	**China**	**PX656674**	**PX656682**	**PX676542**	**This study**
*G. corollinum*	Herb. Sunhede 7744	Sweden	KF988360	KF988482	KF988617	Zamora et al. 2014a
*G. corollinum*	MA-Fungi 5746	Spain	KF988359	KF988481	KF988616	Zamora et al. 2014a
*G. coronatum*	PRM:842868	Hungary	KP687495	KP687456	KP687537	Zamora et al. 2015
*G. coronatum*	Zamora 181	Spain	KP687496	KP687457	KP687538	Zamora et al. 2015
*G. courtecuissei*	LIP:PAM/GUAD 11.04	Guadeloupe	MH635002	—	—	Accioly et al. 2019
*G. courtecuissei*	LIP:FH2004090503	Guadeloupe	MH635003	MH635033	—	Accioly et al. 2019
*G. dolomiticum*	FP20151027	Hungary	MT569467	MT569458	MT572905	Unpublished
*G. dolomiticum*	FP20151015	Hungary	MT569464	MT569456	MT572904	Unpublished
*G. elegans*	Herb. Zamora 189	Spain	KF988366	KF988488	KF988623	Zamora et al. 2014a
*G. elegans*	UPS F-560810	Sweden	KF988367	KF988489	KF988624	Zamora et al. 2014a
*G. fibulatum*	CLZhao 36066	China	PQ484151	PQ481919	PQ811828	Yang et al. 2025
*G. fibulatum*	CLZhao 36068	China	PQ484153	PQ481921	PQ811830	Yang et al. 2025
*G. fimbriatum*	Herb. Zamora 234	Spain	KF988369	KF988491	KF988626	Zamora et al. 2014a
*G. fimbriatum*	Herb. Sunhede 7739	Sweden	KF988370	KF988492	KF988627	Zamora et al. 2014a
*G. floriforme*	Herb. Zamora 453	Spain	KF988373	KF988495	KF988630	Zamora et al. 2014a
*G. floriforme*	MA-Fungi 69173	Spain	KF988372	KF988494	KF988629	Zamora et al. 2014a
*G. fornicatum*	Herb. Zamora 255	Spain	KF988374	KF988496	KF988631	Zamora et al. 2014a
*G. fornicatum*	MA-Fungi 30749	Spain	KF988375	KF988497	KF988632	Zamora et al. 2014a
*G. fuscogleba*	NY Trappe 1071	USA	KF988376	KF988498	KF988633	Zamora et al. 2014a
*G. fuscogleba*	FDS-CA-02274	USA	PP883535	—	—	Unpublished
*G. fuscogleba*	NY Trappe 9500	USA	KF988377	KF988499	KF988634	Zamora et al. 2014a
*G. glaucescens*	MA-Fungi 83762	Argentina	KF988378	KF988500	KF988635	Zamora et al. 2014a
*G. glaucescens*	MA-Fungi 83763	Argentina	KF988379	KF988501	KF988636	Zamora et al. 2014a
*G. gorgonicum*	MA:Fungi:92116	Cape Verde	MN754046	MN754084	—	Unpublished
*G. gorgonicum*	MA:Fungi:92118	Cape Verde	MN754045	MN754083	—	Unpublished
*G. hansagiense*	GBL1	Hungary	MN582753	—	—	Unpublished
*G. hariotii*	MA-Fungi 83765	Argentina	KF988381	KF988504	KF988639	Zamora et al. 2014a
*G. hieronymi*	MA-Fungi 83766	Argentina	KF988384	KF988508	KF988643	Zamora et al. 2014a
*G. hieronymi*	MA-Fungi 83767	Argentina	KF988344	KF988509	KF988644	Zamora et al. 2014a
*G. hirsutum*	MA:Fungi:67886	Brazil	MH538295	—	—	Accioly et al. 2019
*G. hirsutum*	LIP:MR/GUY 12.171	French Guiana	MH635004	—	—	Accioly et al. 2019
*G. hungaricum*	Sunhede 5993	Czech Republic	KP687500	KP687461	KP687542	Zamora et al. 2015
*G. javanicum*	TNS:TKG-GE-90902	Japan	JN845100	JN845218	—	Kasuya et al. 2012
*G. juliae*	LE-F 335197	Russia	OM935687	—	—	Rebriev et al. 2023
*G. juliae*	LE-F 335198	Russia	OM935688	—	—	Rebriev et al. 2023
*G. kuharii*	MA:Fungi:86913	Argentina	KP687502	KP687463	KP687544	Zamora et al. 2015
*G. kuharii*	MA-Fungi 83795	Argentina	KF988463	KF988598	KF988733	Zamora et al. 2014a
***G. kunyuense***	***FFAAS3413* Holotype**	**China**	**PX656677**	**PX656685**	**—**	**This study**
***G. kunyuense***	***FFAAS3414***	**China**	**PX656678**	**PX656686**	**—**	**This study**
***G. kunyuense***	***FFAAS3415***	**China**	**PX656679**	**PX656687**	**—**	**This study**
*G. lageniforme*	Herb. Zamora 207	Spain	KF988388	KF988513	KF988648	Zamora et al. 2014a
*G. lageniforme*	Herb. Zamora 316	Spain	KF988339	KF988514	KF988649	Zamora et al. 2014a
*G. laneum*	HMJAU65711	China	OP964640	OP964638	—	Wang et al. 2023
*G. laneum*	HMJAU65704	China	OP964641	OP964639	—	Wang et al. 2023
*G. marginatum*	ERRO 2012112609	Spain	KP687504	KP687465	KP687546	Zamora et al. 2015
*G. marginatum*	PRM:842884	Czech Republic	KP687507	KP687468	KP687549	Zamora et al. 2015
*G. melanocephalum*	Herb. Sunhede 7737	Sweden	KF988396	KF988523	KF988658	Zamora et al. 2014a
*G. melanocephalum*	Herb. Zamora 34	Spain	KF988395	KF988522	KF988657	Zamora et al. 2014a
*G. melanorhynchum*	HMJAU65765	China	OP964616	—	—	Wang et al. 2023
*G. melanorhynchum*	HMJAU65768	China	OP964618	OP964615	—	Wang et al. 2023
*G. meridionale*	AVM 2708	Spain	KP687508	KP687469	KP687550	Zamora et al. 2014a
*G. meridionale*	Herb. Zamora 252	Spain	KF988412	KF988540	KF988675	Zamora et al. 2015
*G. michelianum*	Herb. Sunhede 7738	Sweden	KF988397	KF988524	KF988659	Zamora et al. 2014a
*G. michelianum*	Herb. Zamora 227	Spain	KF988398	KF988525	KF988660	Zamora et al. 2014a
*G. microphole*	HMJAU65720	China	OP964636	OP964643	—	Wang et al. 2023
*G. microphole*	HMJAU65721	China	OP964637	OP964644	—	Wang et al. 2023
*G. minimum*	Herb. Sunhede 7746	Sweden	KF988401	KF988529	KF988664	Zamora et al. 2014a
*G. minimum*	Herb. Zamora 191	Spain	KF988400	KF988528	KF988663	Zamora et al. 2014a
*G. minutisporum*	CORD15	Argentina	KM260665	—	—	Caffot et al. 2016
*G. minutisporum*	CORD14	Argentina	KM260664	—	—	Caffot et al. 2016
*G. mirabile*	TNS:KH-JPN10-701	Japan	JN845107	JN845225	—	Kasuya et al. 2012
*G. mirabile*	TNS:KH-JPN10-714	Japan	JN845109	JN845227	—	Kasuya et al. 2012
*G. mongolicum*	HMJAU65762	China	OP964647	OP964645	—	Wang et al. 2023
*G. mongolicum*	HMJAU65763	China	OP964648	OP964646	—	Wang et al. 2023
*G. morganii*	Herb. Lebeuf HRL0177	Canada	KF988406	KF988534	KF988669	Zamora et al. 2014a
*G. neoamericanum*	UFRN:Fungos:2302	Brazil	MH635001	MH635040	—	Accioly et al. 2019
*G. neoamericanum*	LIP:JLC12030103	French Guiana	MH635014	MH635038	—	Accioly et al. 2019
*G. oxysepalum*	HMJAU65727	China	OP964632	OP964622	—	Wang et al. 2023
*G. oxysepalum*	HMJAU65730	China	OP964629	—	—	Wang et al. 2023
*G. papinuttii*	MA:Fungi:86912	Argentina	KP687515	KP687477	KP687558	Zamora et al. 2015
*G. papinuttii*	MA-Fungi 83764	Argentina	KF988380	KF988502	KF988637	Zamora et al. 2015
*G. parvisporum*	BAFC:51926	Argentina	MG196037	MG196035	MG196038	Zamora et al. 2017
*G. parvisporum*	MA-Fungi 83793	Argentina	KF988461	KF988596	KF988731	Zamora et al. 2014a
*G. parvistriatum*	JCZ 285	Spain	JN943161	JN939571	JN991282	Unpublished
*G. parvistriatum*	JCZ 272	Spain	JN943162	JN939572	JN991283	Unpublished
*G. pectinatum*	MA:Fungi:28156	Spain	KP687516	KP687478	KP687559	Zamora et al. 2015
*G. pectinatum*	UPS:F-149330	Sweden	KP687519	KP687481	KP687562	Zamora et al. 2015
*G. pleosporum*	MA-Fungi 56971	Cameroon	KF988416	KF988544	KF988679	Zamora et al. 2014a
*G. pseudolimbatum*	Herb. Zamora 231	Spain	KF988419	KF988547	KF988682	Zamora et al. 2014a
*G. pseudolimbatum*	UPS F-560804	Sweden	KF988420	KF988548	KF988683	Zamora et al. 2014a
*G. pseudosaccatum*	HMJAU65778	China	OP964624	—	—	Wang et al. 2023
*G. pseudosaccatum*	HMJAU65769	China	OP964628	OP964634	—	Wang et al. 2023
*G. pseudostriatum*	MJ5738	Sweden	KC581996	KC581996	—	Jeppson et al. 2013
*G. pseudostriatum*	MJ8812	Sweden	KC758595	KC758595	—	Jeppson et al. 2013
*G. pusillipilosum*	UFRN:Fungos 2759	Brazil	KX761177	KX761178	—	Unpublished
*G. pusillipilosum*	UFRN:Fungos 2315	Brazil	KX761175	KX761176	—	Unpublished
*G. quadrifidum*	Herb. Zamora 170	Spain	KF988421	KF988549	KF988684	Zamora et al. 2014a
*G. quadrifidum*	Zamora 300	Spain	KP687524	KP687486	KP687567	Zamora et al. 2015
*G. rubellum*	UFRN:Fungos:2844	Brazil	MH634999	MH635031	—	Accioly et al. 2019
*G. rubropusillum*	UFRN:Fungos:2308	Brazil	MH634994	MH635027	—	Accioly et al. 2019
*G. rufescens*	Herb. Zamora 274	Spain	KF988425	KF988553	KF988688	Zamora et al. 2014a
*G. rufescens*	Herb. Zamora 253	Spain	KF988424	KF988552	KF988687	Zamora et al. 2014a
*G. saccatum*	Herb. Zamora 461	Spain	KF988431	KF988561	KF988696	Zamora et al. 2014a
*G. saccatum*	Herb. Zamora 260	Spain	KF988430	KF988560	KF988695	Zamora et al. 2014a
*G. sanglinense*	HMSAU15020	China	OP050116	OP050161	—	Wu et al. 2024
*G. sanglinense*	HMSAU15025	China	OP050120	OP050165	—	Wu et al. 2024
*G. sanglinense*	HMSAU15023	China	OP050118	OP050163	—	Wu et al. 2024
*G. sanglinense*	HMSAU15024	China	OP050119	OP050164	—	Wu et al. 2024
*G. schmidelii*	MCVE 25701	Switzerland	PQ302313	—	—	Unpublished
*G. schmidelii*	Herb. Zamora 279	Spain	KF988434	KF988564	KF988699	Zamora et al. 2014a
*G. sinense*	CLZhao 36033	China	PQ484158	PQ481924	PQ822019	Yang et al. 2025
*G. sinense*	CLZhao 36031	China	PQ484156	PQ481930	PQ822017	Yang et al. 2025
*G. smardae*	TSNilsen	Spain	KC581976	KC581976	—	Jeppson et al. 2013
*G. striatum*	S:F-46048	Sweden	JN845124	JN845242	—	Kasuya et al. 2012
*G. striatum*	MJ8807	Sweden	KC581960	KC581960	—	Jeppson et al. 2013
*G. tenuipes*	CANB:775658	Australia	KP687527	KP687489	KP687571	Zamora et al. 2015
*G. tenuipes*	CANB:738350	Australia	KP687526	KP687488	KP687570	Zamora et al. 2015
*G. trachelium*	CLZhao 35992	China	PQ484161	PQ481927	PQ783783	Yang et al. 2025
*G. trachelium*	CLZhao 36055	China	PQ484162	PQ481928	PQ783784	Yang et al. 2025
*G. triplex*	UPS F-014630 (213863)	Madagascar	KF988444	KF988578	KF988713	Zamora et al. 2014a
*G. triplex*	MA-Fungi 83784	Argentina	KF988445	KF988579	KF988714	Zamora et al. 2014a
*G. velutinum*	MA-Fungi 83786	Argentina	KF988447	KF988582	KF988717	Zamora et al. 2014a
*G. velutinum*	MA-Fungi 83785	Argentina	KF988446	KF988581	KF988716	Zamora et al. 2014a
***G. villopannosum***	***FFAAS3411* Holotype**	**China**	**PX656675**	**PX656683**	**PX676543**	**This study**
***G. villopannosum***	***FFAAS3412***	**China**	**PX656676**	**PX656684**	**PX676544**	**This study**
*G. violaceum*	MA-Fungi 82487	Argentina	KF988451	KF988586	KF988721	Zamora et al. 2014a
*G. violaceum*	BAFC 51671	Argentina	KF988450	KF988585	KF988720	Zamora et al. 2014a
*G. wrightii*	MLHC 526	Argentina	MK732527	MK732527	MK732534	Unpublished
*G. wrightii*	MLHC 1903	Argentina	MK732528	MK732528	MK732535	Unpublished
*G. yanshanense*	BJTC 381	China	MZ508878	MZ509383	MZ571175	Zhou et al. 2022
*G. yanshanense*	BJTC 057	China	MZ508879	MZ509384	MZ571176	Zhou et al. 2022
*G. yunnanense*	CLZhao 24893	China	PP511308	PP511310	—	Yang et al. 2024
*G. yunnanense*	CLZhao 24922	China	PP511309	PP511311	—	Yang et al. 2024
*Schenella pityophila*	Herb. Zamora 530	Spain	KF988346	KF988464	KF988599	Zamora et al. 2014a
*Schenella pityophila*	Herb. Zamora 531	Spain	KF988347	KF988465	KF988600	Zamora et al. 2014a

Remarks: Newly generated sequences are emphasized in bold; “—” refers to a missing sequence.

The phylogenetic results revealed that 12 clades were well supported (Fig. 1). *Geastrum kunyuense*, *G. villopannosum*, and *G. capillitiononramosum* were located in Clade 1, Clade 7, and Clade 10, with strong support (*G. kunyuense*: BS/BPP = 97/0.99; *G. villopannosum*: BS/BPP = 100/1.00; *G. capillitiononramosum*: BS/BPP = 100/1.00), respectively. *Geastrum kunyuense* was placed along with six species in Clade 1 with high statistical support (BS/BPP = 100/1.00), namely, *G. michelianum* (Sacc.) W.G. Sm., *G. melanocephalum* (Czern.) V.J. Staněk, *G. fuscogleba* (Zeller) Jeppson & E. Larss., *G. fibulatum* Xin Yang & C.L. Zhao, *G. smardae* V.J. Staněk, and *G. triplex* Jungh. (Staněk 1956; Kasuya et al. 2012; Jeppson et al. 2013; Zamora et al. 2014a; Yang et al. 2025). Within this clade, *G. kunyuense* formed a strongly supported group (BS/BPP = 100/1.00) with *G. smardae* and *G. triplex*. Phylogenetic analysis revealed that *G. kunyuense* was most closely related to *G. smardae*, a species reported from the Northern Hemisphere (Staněk 1956; Jeppson et al. 2013). Clade 7 was comprised of four species: *G. villopannosum*, *G. yunnanense* Xin Yang & C. L. Zhao, *G. velutinum* Morgan, and *G. javanicum* Lév., which were distributed in the Northern Hemisphere (Zhou et al. 2007; Kotlaba et al. 2018; Yang et al. 2024). *Geastrum villopannosum* was more closely related to *G. yunnanense* (BS/BPP = 85/--) than the other two species, which were reported from the United States and Indonesia (Zhou et al. 2007; Kotlaba et al. 2018; Yang et al. 2024). In the phylogenetic tree, *G. capillitiononramosum* and *G. fimbriatum* Fr. exhibited a sister relationship and formed two distinct lineages with high support (BS/BPP = 100/1.00).

### Taxonomy

#### Geastrum capillitiononramosum

Taxon classificationFungiGeastralesGeastraceae

T. Wang, Q. Na & Y. P. Ge sp. nov.

FD65E380-8DA0-5815-A403-0F83FE32523B

861500

Figs 2, 3, 4

##### Diagnosis.

Mycelial layer encrusted with debris, pseudoparenchymatous layer lacking a collar, endoperidial body sessile without an apophysis, and peristome distinctly delimited. Differs from *G. fimbriatum* by distinctly delimited peristome and columnar-ornamented basidiospores.

**Figure 2.**
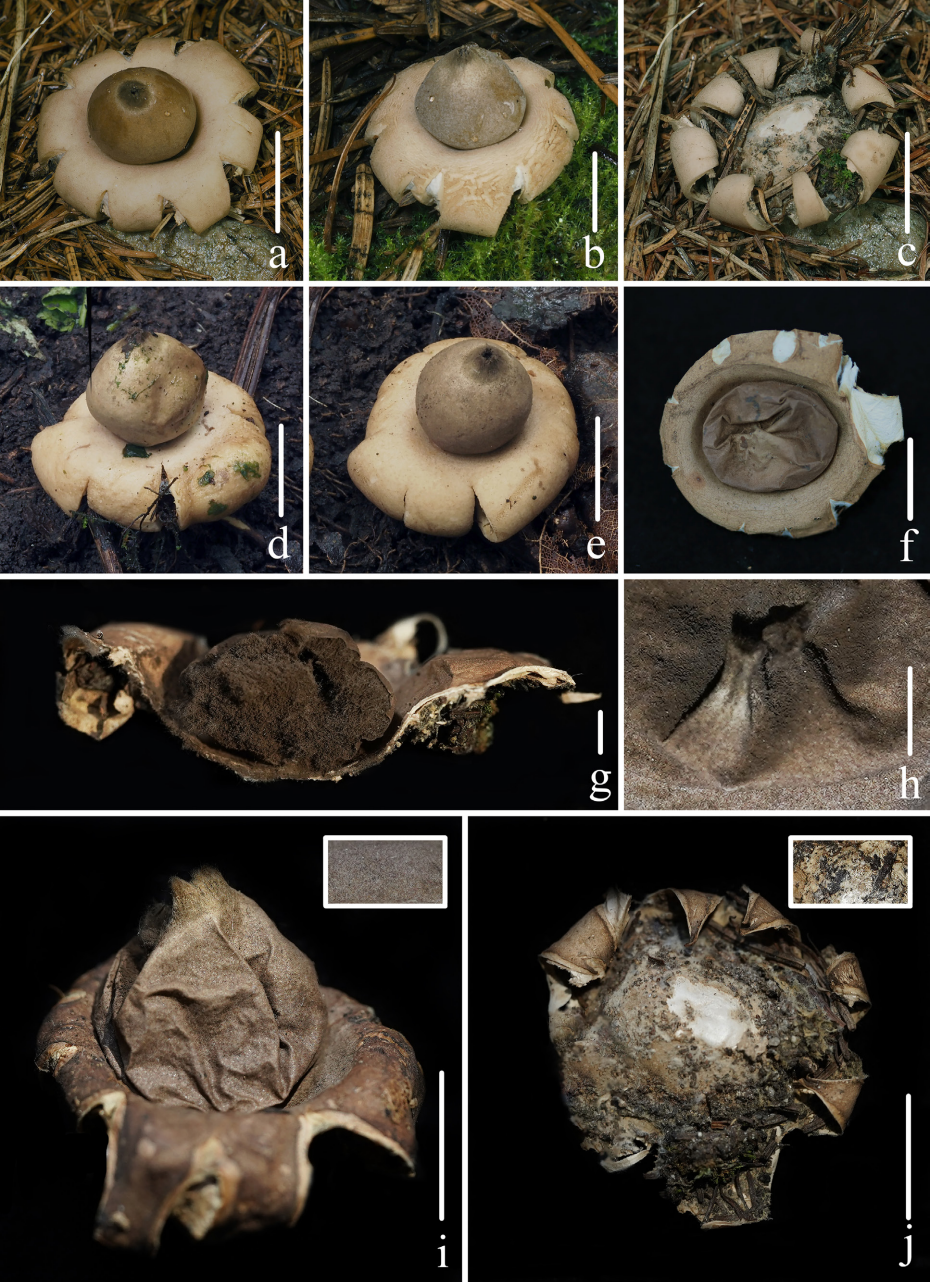
Basidiomata of *Geastrum capillitiononramosum*. **a–c, g, h, j**. *FFAAS3409*; **d, e, i**. *FFAAS3408* (**Holotype**); **f**. *FFAAS3410*. **a–f**. Basidiomata; **g**. Longitudinal section of basidiomata; **h**. Peristome; **i**. Basidiomata and the smooth endoperidium; **j**. Debris. Scale bars: 10 mm (**a–f, i, j**); 3 mm (**g**); 1 mm (**h**). Photos by Yupeng Ge and Tian Wang.

**Figure 3.**
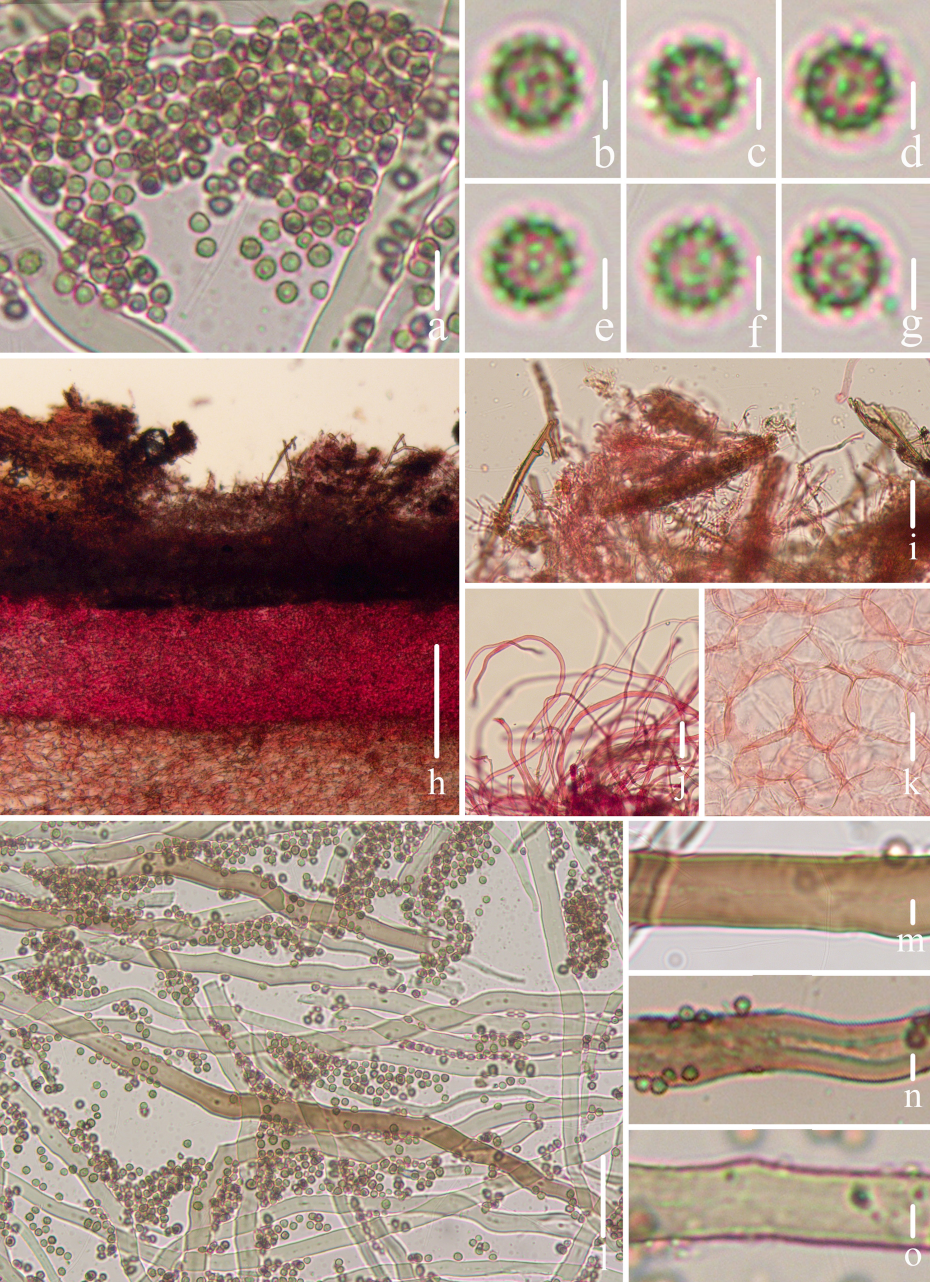
Microscopical features of *Geastrum capillitiononramosum FFAAS3408* (**Holotype**). **a–g**. Basidiospores; **h**. Exoperidium; **i**. Mycelial layer; **j**. Fibrous layer; **k**. Pseudoparenchymatous layer; **l–o**. Capillitial hyphae. Scale bars: 10 μm (**a**); 2 μm (**b–g, m–o**); 300 μm (**h**); 30 μm (**i–l**). Structures **a–g, l–o** were rehydrated in a 5% KOH aqueous solution, and **h–k** were stained in a 1% Congo red aqueous solution.

**Figure 4.**
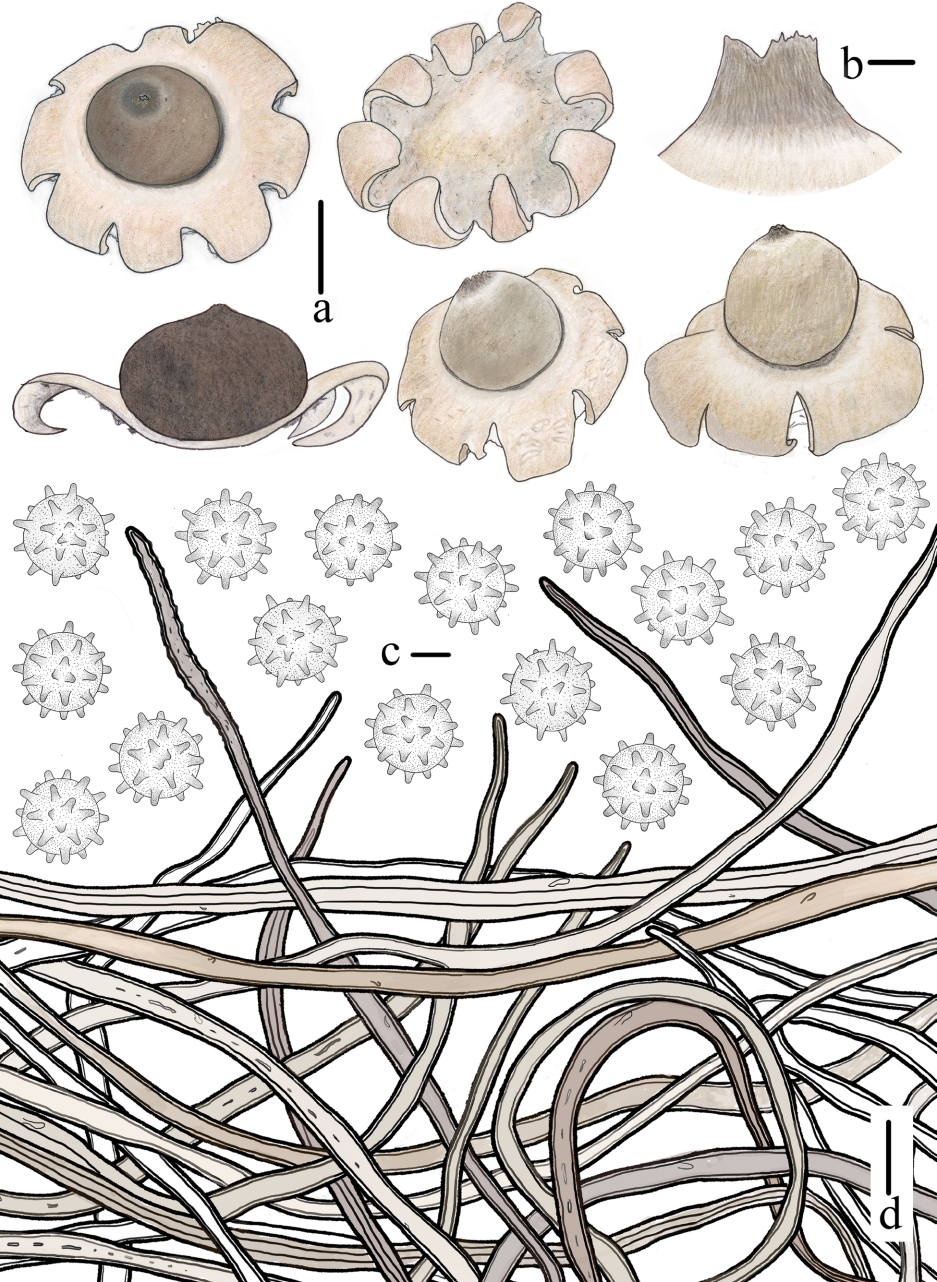
Morphological features of *Geastrum capillitiononramosum FFAAS3408* (**Holotype**). **a**. Basidiomata and longitudinal section; **b**. Peristome; **c**. Basidiospores; **d**. Capillitial hyphae. Scale bars: 10 mm (**a**); 2 mm (**b**); 2 μm (**c**); 20 μm (**d**). Drawing by Tian Wang.

##### Holotype.

China • Heilongjiang Province, Mudanjiang City, Mudanfeng National Forest Park, 23 August 2024, leg. Tian Wang, Yupeng Ge, Qin Na, Zengcai Liu, Ruipeng Liu, Pengyu Du, and Ying Yu, 525 m asl, *FFAAS3408* (collection no. NJ6629).

##### Etymology.

From Latin *capillitium* and *non-ramosus*, referring to the unbranched capillitial hyphae.

##### Description.

***Expanded basidiomata*** 14–32 mm in diameter, shallowly saccate to arched. ***Exoperidium*** splitting into 6–9 lobes, lobes 3–14 mm wide, mostly broad but occasionally narrow, gradually narrowing towards the apex, revolute outward under the exoperidial disc, non-hygroscopic, thin when dry. ***Pseudoparenchymatous layer*** Pale Olive-Buff (XL21’’’f) or Ivory Yellow (XXX21’’f), smooth, persistent, lacking a collar. ***Fibrous layer*** Pale Olive-Buff (XL21’’’f), leathery, firmly attached to pseudoparenchymatous layer. ***Mycelial layer*** *Olive-Buff (XL21’’’d) to Deep Olive-Buff (XL21’’’b), with fugacious debris. ***Endoperidial body*** 7–15 mm in diameter, globose to subglobose, sessile, without an apophysis. ***Endoperidium*** Deep Olive-Buff (XL21’’’b) to Citrine-Drab (XL21’’’i) or Pale Smoke Gray (XLVI21’’’’f) to Light Grayish Olive (XLVI21’’’’b), smooth. ***Peristome*** shortly and broadly conical, fibrillose or silky fibrillose, Deep Grayish Olive (XLVI21’’’’i), the same color as or darker than endoperidium, distinctly delimited.

***Basidiospores*** [100/4/3] 2.9–***3.1***–3.2 × 2.9–***3.1***–3.2 μm, [Q = 1.00–1.02, Qm = ***1.01*** ± 0.01] [holotype (50/2/1) 3.0–***3.1***–3.2 × (2.8) 3.0–***3.1***–3.2 μm, Q = 1.00–1.02, Qm = ***1.01*** ± 0.01], globose, yellowish brown to dark brown in 5% KOH aqueous solution, with regularly columnar ornamentation, 0.5–0.7 μm in length (under oil). ***Capillitial hyphae*** 3.7–7.7 μm in diameter, rarely up to 9.0–11.5 μm, apically attenuated, unbranched, light brown to yellowish brown, mostly hyaline, with sparse debris, very few granular protuberances, thick-walled (0.5–0.9 μm). ***Pseudoparenchymatous layer*** made up of 35–84 × 22–64 μm cells, ellipsoid, oblong or angular, pale yellowish, hyaline, thick-walled (0.6–1.3 μm). ***Fibrous layer*** made up of hyphae measuring 5.3–7.9 μm in diameter, pale yellowish to yellowish brown, hyaline, thick-walled (0.5–0.9 μm). ***Mycelial layer*** made up of hyphae measuring 2.9–4.7 μm in diameter, yellowish brown to dark brown, thick-walled (0.6–0.8 μm), with occasionally observed clamps.

##### Habit and habitat.

Scattered on the ground or humus layer in secondary *Quercus mongolica* Fisch. ex Ledeb. Forest, mainly under trees of *Quercus*, *Syringa*, and *Fraxinus*.

##### Known distribution.

Heilongjiang Province, Jilin Province, China.

##### Additional specimens examined.

China • Heilongjiang Province, Hegang City, Luobei County, Taipinggou National Nature Reserve, 30 August 2023, leg. Tian Wang, Yupeng Ge, Qin Na, Renxiu Wei, Menghui Han, Yawei Li, Xinyu Tong, and Jiang Bian, 588 m asl, *FFAAS3409* (collection no. GN2439, Paratype); Jilin Province, Changchun City, Jingyuetan National Scenic Area, 28 July 2019, leg. Yuguang Fan and Tian Wang, 314 m asl, *FFAAS3410* (collection no. GN2726, Paratype).

##### Notes.

*Geastrum capillitiononramosum* and *G. fimbriatum* shared the same fugacious debris, sessile endoperidial body, and fibrillose peristome, but the latter possessed an indistinctly delimited peristome, delicately echinulate or verrucose ornamentation of the basidiospores (under oil), and branched capillitial hyphae (Tai 1979; Zhou et al. 2007; Trierveiler-Pereira et al. 2009; Jeppson et al. 2013; Karun et al. 2014; Zamora et al. 2014a). *Geastrum rufescens* has been reported from Europe, Asia, Africa, Oceania, and North America (Coker 1924; Tai 1979; Pegler et al. 1995; Zhou et al. 2007). *Geastrum capillitiononramosum* resembled *G. rufescens* in the presence of an encrustation of debris, the absence of an apophysis, and a fibrillose to silky fibrillose peristome (Coker 1924; Tai 1979; Pegler et al. 1995; Zhou et al. 2007; Jeppson et al. 2013; Kuhar et al. 2013; Zamora et al. 2014a). However, *G. rufescens* differed in having a collar, larger basidiospores (3.6–3.8 μm in diam., Dissing and Lange 1962; 5.0–6.0 μm in diam., Sunhede 1989), and branched capillitial hyphae (Coker 1924; Tai 1979; Pegler et al. 1995; Zhou et al. 2007; Jeppson et al. 2013; Kuhar et al. 2013; Zamora et al. 2014a). *Geastrum capillitiononramosum* may be confused with *G. setiferum* Baseia (holotype collected from Brazil) due to its possession of a sessile endoperidial body, similarly sized basidiospores (2.5–3.0 μm in diam.), and unbranched capillitial hyphae, but *G. setiferum* differs from *G. capillitiononramosum* by its squamose mycelial layer, setose endoperidium, and habitat near *Chloroleucon foliolosum* (Benth.) G.P. Lewis and *Jacaranda cuspidifolia* Mart. (Baseia et al. 2002).

#### Geastrum villopannosum

Taxon classificationFungiGeastralesGeastraceae

T. Wang, Q. Na & Y. P. Ge sp. nov.

6D64ABB2-9069-53E2-9634-734293B7EC53

861501

Figs 5, 6, 7

##### Diagnosis.

Mycelial layer felted villus, encrusted with debris; pseudoparenchymatous layer lacking a collar; endoperidial body sessile without an apophysis; and peristome distinctly delimited. Differs from *G. rubropusillum* J.O. Sousa, Accioly, M.P. Martín & Baseia by larger expanded basidiomata and smaller basidiospores.

**Figure 5.**
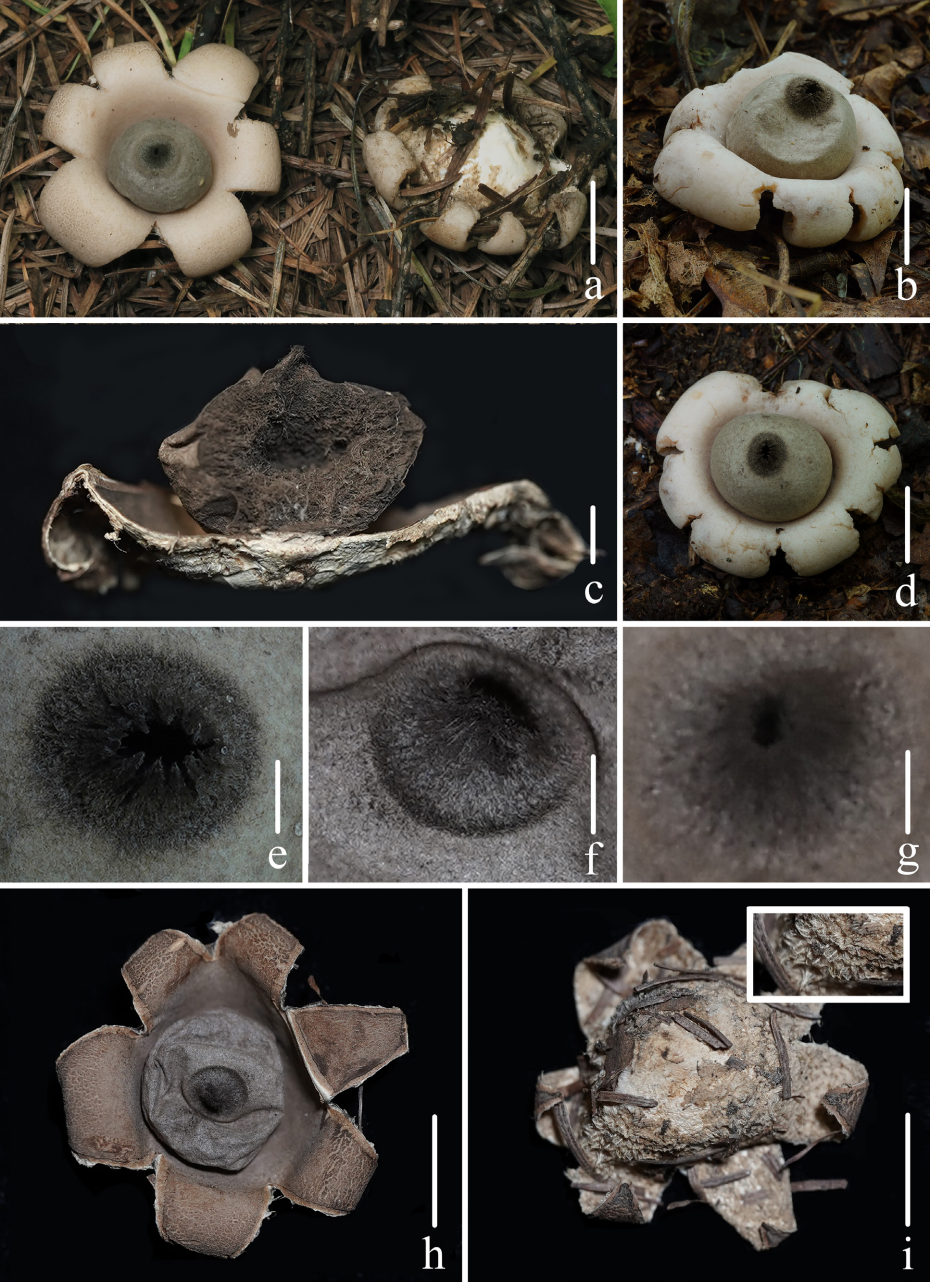
Basidiomata of *Geastrum villopannosum*. **a, c, f–i**. *FFAAS3411* (**Holotype**); **b, d, e**. *FFAAS3412*. **a, b, d**. Basidiomata; **c**. Longitudinal section of basidiomata; **e–g**. Peristome; **h**. Basidiomata and the smooth endoperidium; **i**. Debris. Scale bars: 10 mm (**a, b, d, h, i**); 2 mm (**c, e–g**). Photos by Yupeng Ge and Tian Wang.

**Figure 6.**
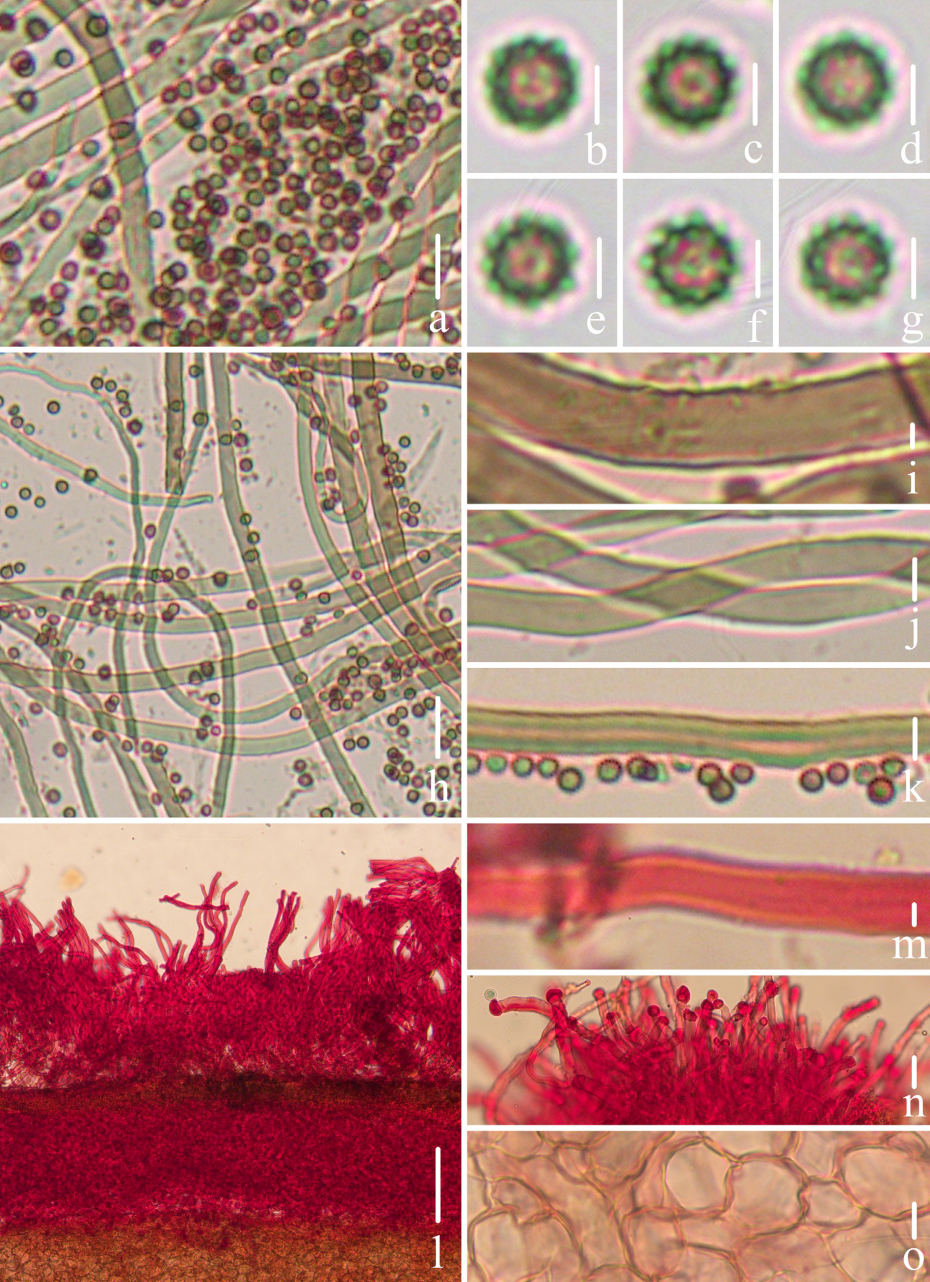
Microscopical features of *Geastrum villopannosum FFAAS3411* (**Holotype**). **a–g**. Basidiospores; **h–k**. Capillitial hyphae; **l**. Exoperidium; **m**. Mycelial layer; **n**. Fibrous layer; **o**. Pseudoparenchymatous layer. Scale bars: 10 μm (**a, j–k, n–o**); 20 μm (**h**); 3 μm (**b–g, i, m**); 100 μm (**l**). Structures **a–k** were rehydrated in a 5% KOH aqueous solution, and **l–o** were stained in a 1% Congo red aqueous solution.

**Figure 7.**
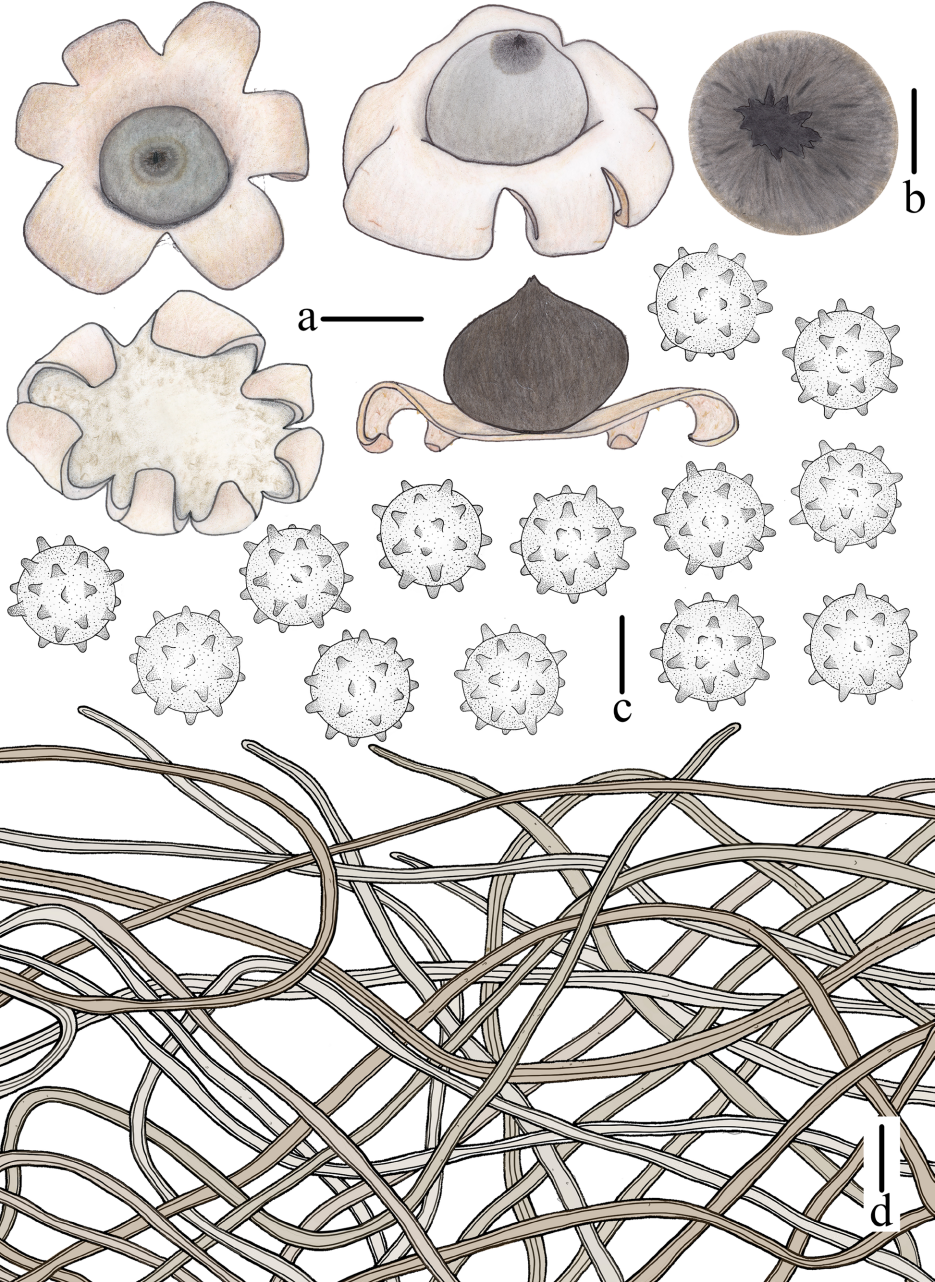
Morphological features of *Geastrum villopannosum FFAAS3411* (**Holotype**). **a**. Basidiomata and longitudinal section; **b**. Peristome; **c**. Basidiospores; **d**. Capillitial hyphae. Scale bars: 10 mm (**a**); 2 mm (**b**); 2 μm (**c**); 15 μm (**d**). Drawing by Tian Wang.

##### Holotype.

China • Heilongjiang Province, Mudanjiang City, Mudanfeng National Forest Park, 24 August 2024, leg. Tian Wang, Yupeng Ge, Qin Na, Zengcai Liu, Ruipeng Liu, Pengyu Du, and Ying Yu, 367 m asl, *FFAAS3411* (collection no. NJ6699).

##### Etymology.

From Latin villus (hairy) and pannosus (felted), referring to the felted villose exoperidium.

##### Description.

***Expanded basidiomata*** 26–38 mm in diameter, shallowly saccate. ***Exoperidium*** splitting into 6–8 lobes, lobes 8–11 mm wide, gradually narrowing towards the apex, acuminate or acute at the lobe apices, revolute outward under the exoperidial disc, non-hygroscopic, thin and brittle when dry. ***Pseudoparenchymatous layer*** Pallid Mouse Gray (LI15’’’’’f), Pallid Quaker Drab (LI1’’’’’f) to Pale Olive-Buff (XL21’’’f), smooth, persistent, lacking a collar. ***Fibrous layer*** Pallid Quaker Drab (LI1’’’’’f) to Pale Olive-Buff (XL21’’’f), firmly attached to pseudoparenchymatous layer. ***Mycelial layer*** Pale Olive-Buff (XL21’’’f) to *Olive-Buff (XL21’’’d), finely felted villus, encrusted with debris. ***Endoperidial body*** 12–21 mm in diameter, globose to subglobose, sessile, without an apophysis. ***Endoperidium*** Light Mineral Gray (XLVII25’’’’f), Mineral Gray (XLVII25’’’’d) to Tea Gray (XLVII25’’’’b), smooth. ***Peristome*** shortly and broadly conical, fibrillose, *Olive-Gray (LI23’’’’’b) to Deep Grayish Olive (XLVI21’’’’i), darker than the endoperidium, distinctly delimited.

***Basidiospores*** [75/3/2] 2.6–***2.7***–2.9 (3.1) × 2.6–***2.7***–2.9 μm, [Q = 1.00–1.02 (1.06), Qm = ***1.01*** ± 0.01] [holotype (50/2/1) 2.6–***2.7***–2.9 × 2.6–***2.7***–2.9 μm, Q = 1.00–1.02, Qm = ***1.01*** ± 0.01], globose to subglobose, yellowish brown to dark brown in 5% KOH aqueous solution, with regularly columnar ornamentation, 0.3–0.5 μm in length (under oil). ***Capillitial hyphae*** 4.1–11.7 μm in diameter, apically attenuated, unbranched, yellowish brown to dark brown, mostly hyaline, with sparse debris, very few granular protuberances, thick-walled (0.4–0.7 μm). ***Pseudoparenchymatous layer*** made up of 16–50 × 15–38 μm cells, ellipsoid, oblong or angular, pale yellowish, hyaline, thick–walled (0.5–0.7 μm). ***Fibrous layer*** composed of hyphae measuring 4.9–9.3 μm in diameter, pale yellowish to yellowish brown, hyaline, thick-walled (0.5–0.7 μm). ***Mycelial layer*** made up of hyphae measuring 4.1–6.5 μm in diameter, yellowish brown to dark brown, thick-walled (0.5–0.8 μm), with occasionally observed clamps.

##### Habit and habitat.

Scattered on the ground or humus layer in secondary *Quercus mongolica* Fisch. ex Ledeb. Forest, mainly under trees of *Quercus*, *Syringa*, *Fraxinus*, *Betula*, and *Tilia*.

##### Known distribution.

Heilongjiang Province, China.

##### Additional specimens examined.

China • Heilongjiang Province, Hegang City, Luobei County, Taipinggou National Nature Reserve, 29 August 2023, leg. Tian Wang, Yupeng Ge, Qin Na, Renxiu Wei, Menghui Han, Yawei Li, Xinyu Tong, and Jiang Bian, 633 m asl, *FFAAS3412* (collection no. GN2317, Paratype).

##### Notes.

*Geastrum villopannosum* was similar to *G. rubropusillum* macroscopically. Both had the same basidiomata coloration, a sessile endoperidial body, and a distinctly delimited peristome, but *G. rubropusillum* could be distinguished by its smaller mature basidiomata (2.9–7.0 × 7.0–9.5 mm), larger basidiospores (3.8–5.9 × 3.7–5.3 μm), and branched capillitial hyphae (Accioly et al. 2019). *Geastrum courtecuissei* P.-A. Moreau & Lécuru, which was found in Guadeloupe, also resembled *G. villopannosum* in the color of the pseudoparenchymatous layer, number of exoperidium lobes (4–8 lobes), and sessile endoperidial body (Accioly et al. 2019). However, *G. courtecuissei* differed in having a white tomentum endoperidium and larger basidiospores (3.8–5.0 × 3.7–4.9 μm) (Accioly et al. 2019). *Geastrum javanicum* Lév. and *G. villopannosum* were well characterized by the felted mycelial layer and unbranched capillitial hyphae, but *G. javanicum* differed from *G. villopannosum* by its deeply saccate expanded basidiomata, an indistinctly delimited peristome, and capillitial hyphae with densely granular protuberances (Teng 1963; Dring 1964; Zhou et al. 2007; Leite et al. 2011). *Geastrum villopannosum* and *G. yunnanense*, reported from China, shared similarly sized basidiomata (20–40 mm in diam.), a pseudoparenchymatous layer lacking a collar, and a distinctly delimited peristome (Yang et al. 2024). However, *G. yunnanense* could be easily distinguished based on the mycelial layer without debris, smaller basidiospores (2.0–2.5 × 1.9–2.5 μm), and rarely branched capillitial hyphae (Yang et al. 2024).

#### Geastrum kunyuense

Taxon classificationFungiGeastralesGeastraceae

T. Wang, Q. Na & Y. P. Ge sp. nov.

9C1097AF-FAF3-57DE-9DF3-1DBF1F9E58F9

861502

Figs 8, 9, 10

##### Diagnosis.

Unexpanded basidiomata with a slight protrusion, mycelial layer not encrusted with debris, pseudoparenchymatous layer lacking a collar, endoperidial body sessile without an apophysis, and peristome with distinctly delimited. Differs from *G. smardae* by saccate expanded basidiomata, sessile endoperidial body, and smaller basidiospores.

**Figure 8.**
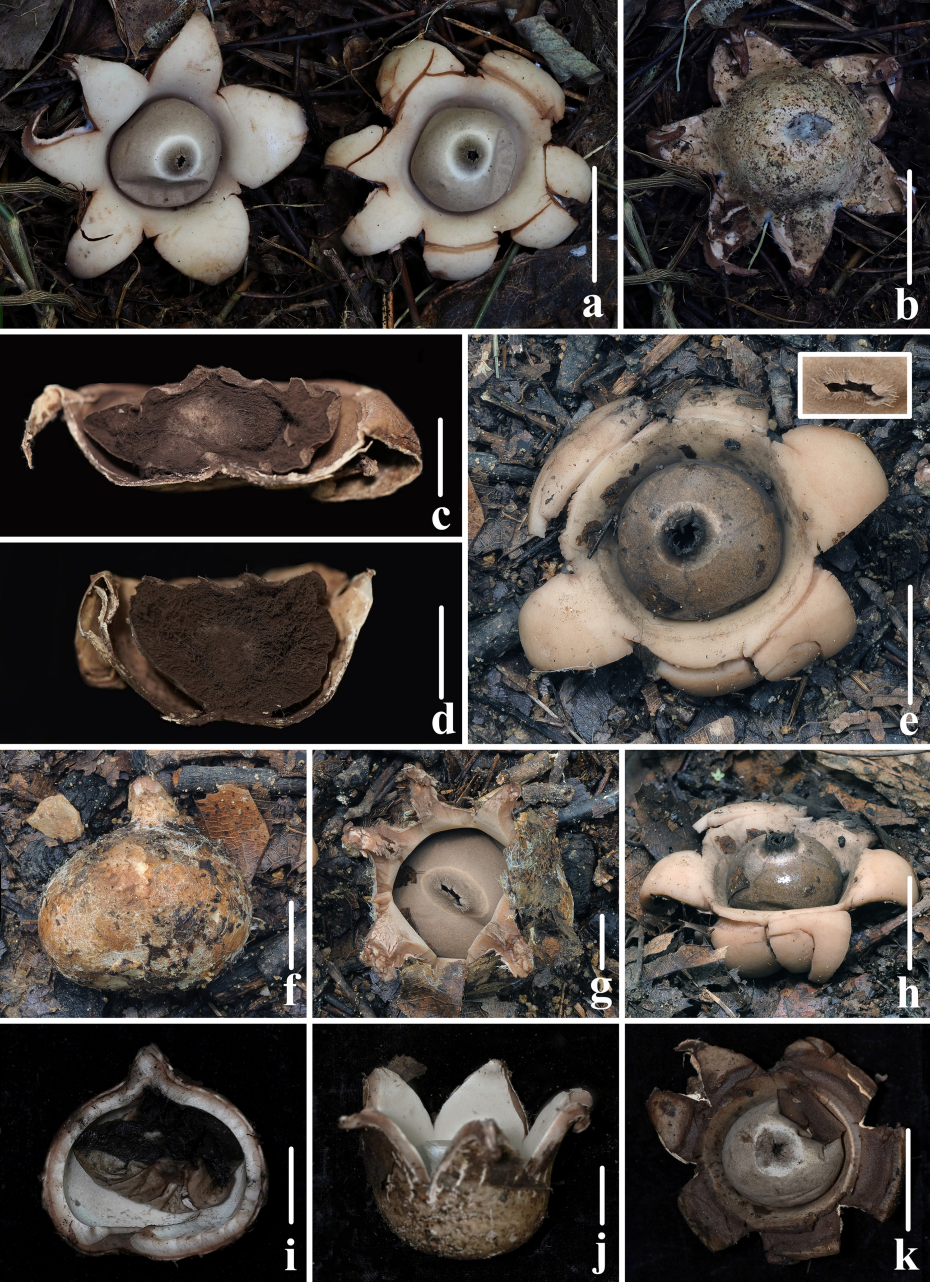
Basidiomata of *Geastrum kunyuense*. **a–c**. *FFAAS3413* (**Holotype**); **d–h**. *FFAAS3414*; **i–k**. *FFAAS3415*. **a, b, e, g, h, j, k**. Expanded basidiomata; **c, d**. Longitudinal section of basidiomata; **f, i**. Unexpanded basidiomata. Scale bars: 20 mm (**a, b, e, h, k**); 10 mm (**c, d, f, g, i, j**). Photos by Yupeng Ge and Tian Wang.

**Figure 9.**
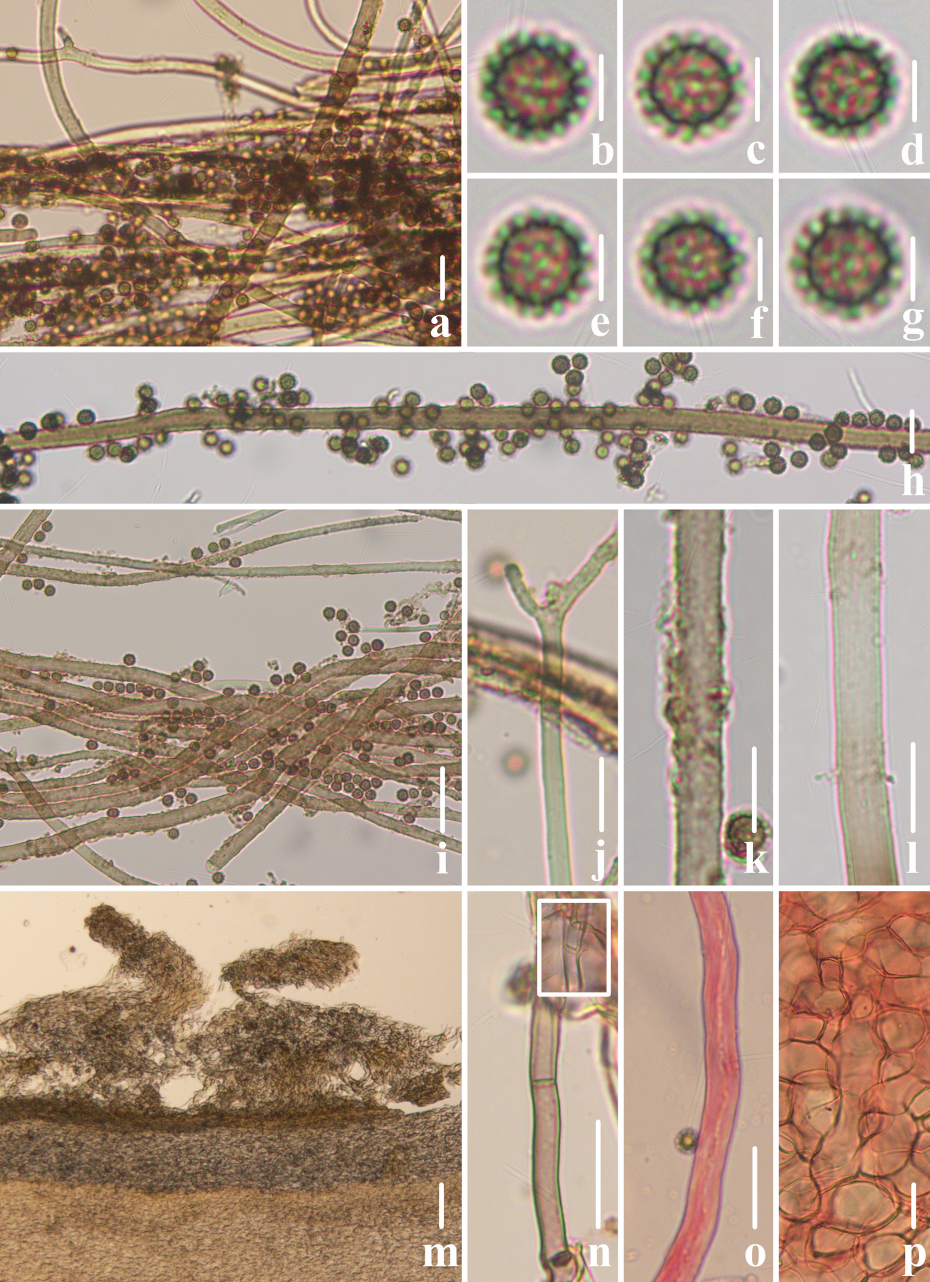
Microscopical features of *Geastrum kunyuense FFAAS3413* (**Holotype**). **a–g**. Basidiospores; **h–k**. Capillitial hyphae; **l**. Exoperidium; **m**. Mycelial layer; **n**. Fibrous layer; **o**. Pseudoparenchymatous layer. Scale bars: 10 μm (**a, j, k, m, n**); 2 μm (**b–g, i**); 30 μm (**h, o**); 100 μm (**l**). Structures **a–k** were rehydrated in a 5% KOH aqueous solution, and **l–o** were stained in a 1% Congo red aqueous solution.

**Figure 10.**
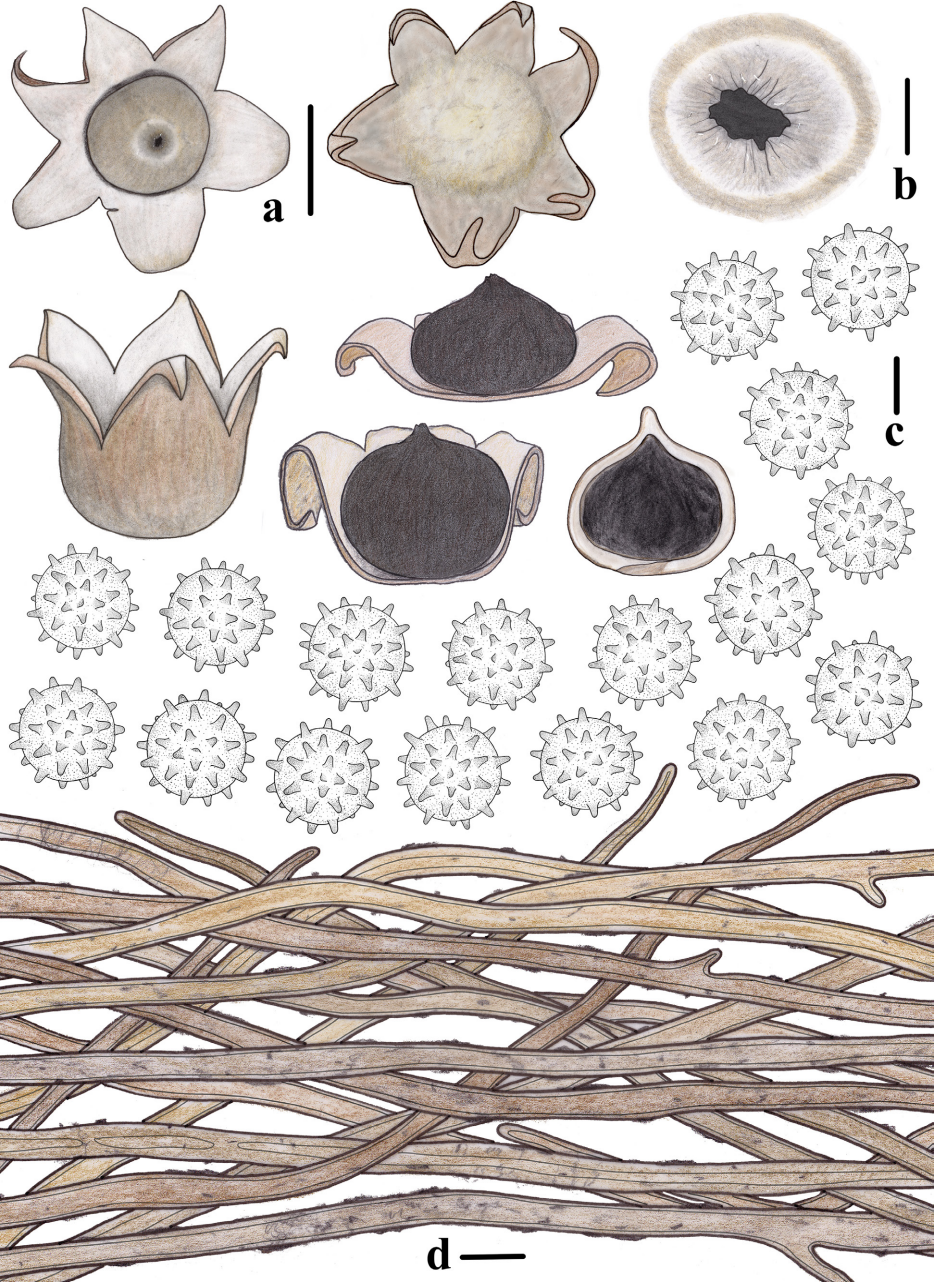
Morphological features of *Geastrum kunyuense FFAAS3413* (**Holotype**). **a**. Basidiomata and longitudinal section; **b**. Peristome; **c**. Basidiospores; **d**. Capillitial hyphae. Scale bars: 20 mm (**a**); 5 mm (**b**); 2 μm (**c**); 10 μm (**d**). Drawing by Tian Wang.

##### Holotype.

China • Shandong Province, Yantai City, Kunyushan National Nature Reserve, 23 August 2019, leg. Tian Wang, Yupeng Ge, Qin Na, Menghui Han, and Renxiu Wei, 505 m asl, *FFAAS3413* (collection no. GN0405).

##### Etymology.

Named after Kunyu Mountain (Shandong Province, China), the type locality where the species was collected.

##### Description.

***Unexpanded basidiomata*** 25–30 mm in diameter, subglobose or onion shaped, with a slight protrusion (4–6 mm high), Cream–Buff (XXX19’’d) to Chamois (XXX19’’b) when fresh, Light Cinnamon-Drab (XLVI13’’’’b) to Light Drab (XLVI17’’’’b) when dry, not encrusted with debris. ***Expanded basidiomata*** 32–61 mm in diameter, shallowly to deeply saccate. ***Exoperidium*** splitting into 5–7 lobes, lobes 6–19 mm wide, gradually narrowing towards the apex, acuminate or acute at the lobe apices, partial lobes revolute outward under the exoperidial disc, non-hygroscopic, thin when dry. ***Pseudoparenchymatous layer*** Cartridge Buff (XXX19’’f) to Ivory Yellow (XXX21’’f) when fresh, Light Drab (XLVI17’’’’b), Light Grayish Olive (XLVI21’’’’b), and Chamois (XXX19’’b) to Deep Grayish Olive (XLVI21’’’’i) when dry, smooth, persistent, lacking a collar. ***Fibrous layer*** Light Brownish Olive (XXX19’’k) to Benzo Brown (XLVI13’’’’i), firmly attached to pseudoparenchymatous layer. ***Mycelial layer*** Cartridge Buff (XXX19’’f) to Ivory Yellow (XXX21’’f) and Marguerite Yellow (XXX23’’f) to Colonial Buff (XXX21’’d) at the lobe apices, not encrusted with debris. ***Endoperidial body*** 22–27 mm in diameter, globose to subglobose, sessile, without an apophysis. ***Endoperidium*** *Pearl Gray (LII35’’’’’f), Light Olive-Gray (LI23’’’’’d) or Pale Smoke Gray (XLVI21’’’’f) to Light Grayish Olive (XLVI21’’’’b), smooth. ***Peristome*** shortly and broadly conical, fibrillose or silky fibrillose, *Pearl Gray (LII35’’’’’f), Light Olive-Gray (LI23’’’’’d) or Dark Olive-Gray (LI23’’’’’i), the same color as or darker than the endoperidium, distinctly delimited, with fine white hairs.

***Basidiospores*** [150/6/3] 3.5–***3.8***–4.1 (4.3) × 3.5–***3.7***–4.0 (4.2) μm, [Q = 1.00–1.03(1.05), Qm = ***1.01*** ± 0.01] [holotype (50/2/1) (3.4)3.6–***3.8***–4.1 (4.2) × 3.5–***3.8***–4.0 (4.2) μm, Q = 1.00–1.03 (1.04), Qm = ***1.01*** ± 0.01], globose, yellowish brown to dark brown in 5% KOH aqueous solution, with regularly columnar ornamentation, 0.4–0.8 μm in length (under oil). ***Capillitial hyphae*** 3.5–6.5 μm in diameter, apically attenuated, shortly branched, light brown to yellowish brown, mostly hyaline, with dense surface debris, densely granular protuberances, thick-walled (0.4–0.8 μm). ***Pseudoparenchymatous layer*** made up of 18–57 × 12–50 μm cells, ellipsoid, oblong or angular, pale yellowish, hyaline, thick-walled (0.5–0.8 μm). ***Fibrous layer*** made up of hyphae measuring 4.9–9.1 μm in diameter, pale yellowish to yellowish brown, hyaline, thick-walled (0.4–0.8 μm). ***Mycelial layer*** made up of hyphae measuring 3.1–7.8 μm in diameter, yellowish brown to dark brown, thick-walled (0.4–0.6 μm), occasionally observed with clamps.

##### Habit and habitat.

Scattered on the ground or humus layer in mixed broadleaf-conifer forests, mainly under trees of *Styphnolobium*, *Populus*, and *Pinus*.

##### Known distribution.

Shandong Province, China.

##### Additional specimens examined.

China • Shandong Province, Tai’an City, Yaoxiang National Forest Park, 21 August 2023, leg. Tian Wang, Yupeng Ge, Qin Na, Menghui Han, and Renxiu Wei, 642 m asl, *FFAAS3414* (collection no. HK1035, Paratype); Shandong Province, Yantai City, Kunyushan National Nature Reserve, 7 September 2024, leg. Tian Wang, Yupeng Ge, Qin Na, and Jingwen Guo, 528 m asl, *FFAAS3415* (collection no. GN2696, Paratype).

##### Notes.

*Geastrum kunyuense* and *G. smardae* shared a similar non-encrusted mycelial layer, smooth endoperidium, and fibrillose peristome, but *G. smardae* differed in arched, expanded basidiomata, a stalked endoperidial body, and larger basidiospores (4.2–5.0 μm in diam.) (Staněk 1958; Jeppson et al. 2013). *Geastrum saccatum* and *G. triplex* resembled *G. kunyuense* in unexpanded basidiomata with a slight protrusion and capillitial hyphae densely covered with debris, but *G. saccatum* could be distinguished by its smaller basidiomata (20–25 mm in diam., Leite et al. 2011; 27.0–29.5 mm in diam., Kuhar et al. 2013), a mycelial layer with fine hair, and smaller basidiospores (2.63–3.16 μm in diam., Kuhar et al. 2013) (Fries 1829; Teng 1963; Sunhede 1989; Leite et al. 2011; Kuhar et al. 2013). The pseudoparenchymatous layer forming a collar and unbranched capillitial hyphae could be used to distinguish *G. triplex* from *G. kunyuense* (Sunhede 1989). *Geastrum pseudosaccatum* T. Bau & Xin Wang and *G. kunyuense* were reported in China, and these two species could be easily confused on account of their similar growth habit, basidiomata coloration, non-encrusted mycelial layer, distinctly delimited peristome, and sessile endoperidial body (Wang et al. 2023). However, *G. pseudosaccatum* differed in its smaller unexpanded basidiomata (12–19 mm in diam.), clustered villi on the unexpanded basidiomata surface, smaller basidiospores (2.6–3.0 μm in diam.), and unbranched capillitial hyphae covered with sparse debris (Wang et al. 2023). *Geastrum fimbriatum* and *G. kunyuense* shared a sessile endoperidial body, a pseudoparenchymatous layer lacking a collar, and similarly sized basidiospores, but *G. fimbriatum* differed from *G. kunyuense* by its mycelial layer encrusted with debris and an indistinctly delimited peristome (Tai 1979; Zhou et al. 2007; Jeppson et al. 2013; Zamora et al. 2014a).

### Key to the species of *Geastrum* in China

**Table:** 

1	Peristome evanescent	***G. melanocephalum***
–	Peristome not evanescent	**2**
2	Endoperidium black	***G. englerianum***
–	Endoperidium not black	**3**
3	Mycelial layer encrusted with debris	**4**
–	Mycelial layer not encrusted with debris	**15**
4	Peristome indistinctly delimited	**5**
–	Peristome distinctly delimited	**9**
5	Endoperidial body with apophysis	**6**
–	Endoperidial body without apophysis	**7**
6	Pseudoparenchymatous layer without a collar	***G. mongolicum***
–	Pseudoparenchymatous layer with a collar	***G. pectinatum***
7	Exoperidium hygroscopic	***G. floriforme***
–	Exoperidium non-hygroscopic	**8**
8	Pseudoparenchymatous layer positive in syringaldazine reaction	**11**
–	Pseudoparenchymatous layer not reacting with syringaldazine	***G. fimbriatum***
9	Peristome fibrillose	**10**
–	Peristome sulcate	**16**
10	Exoperidium splitting into lobes < 5	***G. quadrifidum***
–	Exoperidium splitting into lobes ≥ 5	**12**
11	Pseudoparenchymatous layer gray to dirty white	***G. beijingense***
–	Pseudoparenchymatous layer light red to reddish brown	***G. rufescens***
12	Endoperidial body stalk absent	**13**
–	Endoperidial body stalk present	**14**
13	Expanded basidiomata > 45 mm in diam.	***G. laneum***
–	Expanded basidiomata < 45 mm in diam.	**21**
14	Pseudoparenchymatous layer brown or ferruginous	***G. limbatum***
–	Pseudoparenchymatous layer not brown or ferruginous	***G. minimum***
15	Mycelial pad present	***G. mirabile***
–	Mycelial pad absent	**17**
16	Endoperidium plum-colored	***G. schmidelii***
–	Endoperidium not plum-colored	**18**
17	Pseudoparenchymatous layer with black granular structures	***G. yanshanense***
–	Pseudoparenchymatous layer without black granular structures	**24**
18	Endoperidial body with apophysis	**22**
–	Endoperidial body without apophysis	**19**
19	Exoperidium hygroscopic	***G. campestre***
–	Exoperidium non-hygroscopic	**20**
20	Endoperidial surface rough	**23**
–	Endoperidial surface smooth	***G. elegans***
21	Mycelial layer with felted villus	***G. villopannosum***
–	Mycelial layer without felted villus	***G. capillitiononramosum***
22	Endoperidium with grey granular protrusions	***G. microphole***
–	Endoperidium without grey granular protrusions	***G. striatum***
23	Basidiospores ornamentation 0.4–0.8 μm high	***G. berkeleyi***
–	Basidiospores ornamentation 0.2–0.4 μm high	***G. hariotii***
24	Expanded basidiomata with a stipe	**31**
–	Expanded basidiomata without a stipe	**25**
25	Capillitial hyphae unbranched	**26**
–	Capillitial hyphae branched	**29**
26	Basidiomata growing on living tree	***G. artocarpicola***
–	Basidiomata growing on ground or humus layer	**27**
27	Pseudoparenchymatous layer with a cupulate collar	***G. triplex***
–	Pseudoparenchymatous layer without a cupulate collar	**28**
28	Expanded basidiomata 40–60 mm in diam.	***G. fibulatum***
–	Expanded basidiomata 12–40 mm in diam.	**30**
29	Mycelial layer not dislodged	**33**
–	Mycelial layer easily dislodged	**34**
30	Exoperidium with whitish to cream mycelium	***G. lageniforme***
–	Exoperidium without whitish to cream mycelium	**32**
31	Expanded basidiomata with a stipe 23–35 mm long	***G. suae***
–	Expanded basidiomata with a stipe 5–10 mm long	***G. trachelium***
32	Basidiospores with delicately echinulate ornamentation	***G. oxysepalum***
–	Basidiospores with columnar ornamentation	**36**
33	Pseudoparenchymatous layer not deciduous	**35**
–	Pseudoparenchymatous layer deciduous	**37**
34	Exoperidium hygroscopic	**40**
–	Exoperidium non-hygroscopic	**41**
35	Expanded basidiomata arched	***G. sinense***
–	Expanded basidiomata saccate	**39**
36	Basidiospores 2.6–3.0 μm in diam.	***G. pseudosaccatum***
–	Basidiospores 4.0–5.5 μm in diam.	***G. corollinum***
37	Pseudoparenchymatous layer collar present	***G. morganii***
–	Pseudoparenchymatous layer collar absent	**38**
38	Peristome distinctly delimited	***G. melanorhynchum***
–	Peristome indistinctly delimited	***G. litchi***
39	Expanded basidiomata 12–20 mm in diam.	***G. schweinitzii***
–	Expanded basidiomata 20–40 mm in diam.	**42**
40	Peristome indistinctly delimited	***G. javanicum***
–	Peristome distinctly delimited	***G. hungaricum***
41	Pseudoparenchymatous layer not reacting with syringaldazine	***G. saccatum***
–	Pseudoparenchymatous layer reacting with syringaldazine	***G. velutinum***
42	Basidiospores 3.5–4.1 μm in diam.	***G. kunyuense***
–	Basidiospores 2.6–3.0 μm in diam.	***G. yunnanense***

## Discussion

Morphological characters used in previous infrageneric classifications of *Geastrum* were not always reliably evaluated and were not supported by subsequent phylogenetic analyses (De Toni 1887; Staněk 1958; Dissing and Lange 1962; Ponce de León 1968; Zhou et al. 2007; Zamora et al. 2014a). Both sect. *Geastrum* Pers. and sect. *Schmidelia* J. C. Zamora share similar mycelial layers, pseudoparenchymatous layers, and endoperidial bodies (Persoon 1794; Zamora et al. 2014a). However, phylogenetic analyses showed that sect. *Geastrum* and sect. *Schmidelia* are placed in two different large clades and form two monophyletic groups with high statistical support (BS/BPP = 73/0.89; BS/BPP = 76/1.00), respectively (Persoon 1794; Zamora et al. 2014a). Within the subsections, subsect. *Marginata* P. Ponce de León resembles subsect. *Lageniformia* J.C. Zamora in the pseudoparenchymatous layer not reacting with syringaldazine, a sessile endoperidial body, and a fibrillose and distinctly delimited peristome, but it could be distinguished from subsect. *Lageniformia* by its double-layered mycelial layer (Ponce de León 1968; Zamora et al. 2014a). However, phylogenetic results showed that only one of the three clades of subsect. *Marginata* clusters with subsect. *Lageniformia* with BS/BPP = 100/1.00 (Zamora et al. 2014a). Some species had similar problems; *G. capillitiononramosum* could be easily mistaken for *G. fimbriatum*, but they differed in a key character of the peristome (Jeppson et al. 2013; Karun et al. 2014; Zamora et al. 2014a). In our phylogenetic analyses, *G. capillitiononramosum* clustered with *G. fimbriatum*, forming a clade with high statistical support (BS/BPP = 100/1.00). *Geastrum villopannosum*, *G. yunnanense*, *G. javanicum*, and *G. velutinum* formed a clade with strong support (BS/BPP = 90/1.00). However, a key characteristic of *G. villopannosum* is the debris-encrusted mycelial layer, which is absent in the other three species (Zhou et al. 2007; Kotlaba et al. 2018; Yang et al. 2024).

Species of *Geastrum* from Spain, Sweden, Argentina, Brazil, Germany, Norway, Denmark, and other regions commonly grow on the ground, humus layer, and rotten wood in mixed coniferous and broad-leaved forests, *Picea*, *Pinus*, *Juniperus*, *Quercus*, *Cedrus*, and *Olea*; only a few occur in pastures, abandoned lands, wetlands, bare ground, and sandy areas (Cunningham 1944; Bottomley 1948; Sunhede 1989; Jeppson et al. 2013; Sousa et al. 2015, 2017; Cabral et al. 2017). In China, species of *Geastrum* have also been reported from forests dominated by *Populus*, *Acer*, *Juglans*, *Fraxinus*, and *Larix* (Teng 1963; Liu 1984; Mao 1998; Zhou et al. 2007; Zhou et al. 2022; Wang et al. 2023; Yang et al. 2025).

More than half of the species occur in low-altitude zones (< 1000 m), nearly one-third of the species were found in mid-altitude regions (1000–3500 m), and no species have been documented from high-altitude regions (> 3500 m) (Dissing and Lange 1962; Teng 1963; Sunhede 1989; Mao 1998; Zhou et al. 2007; Hemmes et al. 2011; Leite et al. 2011; Jeppson et al. 2013; Kuhar et al. 2013; Da Silva Alfredo et al. 2016; Wang et al. 2023; Yang et al. 2025). Species of this genus are widely distributed in tropical and temperate regions, with lower diversity in frigid zones abroad, whereas in China they are mainly found in temperate areas and are rarely reported in tropical and frigid regions (Dissing and Lange 1962; Teng 1963; Liu 1984; Sunhede 1989; Hemmes et al. 2011; Leite et al. 2011; Jeppson et al. 2013; Kuhar et al. 2013; Da Silva Alfredo et al. 2016; Zhou et al. 2022; Wang et al. 2023; Wu 2023; Zhang et al. 2023; Wu et al. 2024; Yang et al. 2025).

## Supplementary Material

XML Treatment for Geastrum capillitiononramosum

XML Treatment for Geastrum villopannosum

XML Treatment for Geastrum kunyuense

## Additional information

### Artificial Intelligence (AI) use

The authors accept full responsibility for the content of the manuscript, including the disclosure of any use of AI.

No AI tools were used in the preparation of this manuscript.

### Author ORCIDs

T. Wang https://orcid.org/0009-0004-0573-9530

Q. Na https://orcid.org/0000-0001-8406-6389

L. Zou https://orcid.org/0009-0008-6823-3843

H. Zeng https://orcid.org/0000-0003-2025-844X

Y. Ge https://orcid.org/0000-0001-5754-201X

### Data availability

All of the data that support the findings of this study are available in the main text.
